# Sleep quality in subjects suffering from chronic pain

**DOI:** 10.1007/s00508-017-1256-1

**Published:** 2017-09-07

**Authors:** Mohammad Keilani, Richard Crevenna, Thomas Ernst Dorner

**Affiliations:** 1 Department of Physical Medicine and Rehabilitation, Medical University of Vienna, Waehringer Guertel 18–20, 1090 Vienna, Austria; 2 Department for Social and Preventive Medicine, Centre for Public Health, Medical University of Vienna, Kinderspitalgasse 15/I, 1090 Vienna, Austria

**Keywords:** Chronic pain, Sleep problems, Health-related quality of life

## Abstract

**Background:**

Sleeping problems are very common in patients with chronic pain. The aim of the study was to investigate the association between different dimensions of chronic pain and sleep quality in chronic pain patients.

**Methods:**

In this cross-sectional interview-based questionnaire study, patients from 3 different pain treatment centers in Vienna aged 18–65 years, with pain lasting 3 months or longer were asked to participate. The association between the short-form McGill pain questionnaire (SF-MPQ) and sleep quality (sleep onset latency, interrupted sleep due to pain, sleep duration and recovering effect of sleep) was assessed.

**Results:**

In this study 121 patients (male 32, female 89, mean age 49 ± 9 years) could be analyzed. Of the patients 38.8% needed more than 30 min for falling asleep, 63.6% reported sleep fragmentation, 30.6% slept less than 5 h and 60.3% reported no recovering effect of sleep. The strongest associations between pain characteristics and sleep quality were found for pain intensity and affective pain aspects. Logistic regression analyses revealed that one point more in the total score of SF-MPQ increased the odds of needing more than 30 min for falling asleep, waking up more than 3 times due to pain, sleeping less than 5 h, and perceiving the sleep as non-recovering, by 6%. Adjusting for physical and psychological quality of life lowered the odds ratios and the association was no longer significant.

**Conclusion:**

The results underline the importance of paying attention to sleep quality in patients with chronic pain. The results also indicate that psychological factors might mediate the association between pain and sleep quality.

## Introduction

Chronic pain affects approximately 19% of adults in Europe [1]. In Austria 25% of the adult population is affected by chronic pain [2]. These prevalence rates vary by the types of conditions of chronic pain and rates are higher for women and older adults or subjects with lower educational level [3–5]. Sociodemographic factors, such as sex interact with the mechanisms of coping with chronic pain [2, 6, 7] and therefore play an important role in the consequences of chronic pain. One of the most frequently reported problems for adults living with chronic pain is sleep disturbance [1, 4, 8]. It is notable that approximately two thirds of the general population with chronic pain are less able or unable to sleep because of pain [1]. According to a study carried out in primary care, approximately 40%s of chronic pain patients reported poor sleep quality, where age, female sex, low income, higher pain intensity and the presence of depression were significantly associated with poorer sleep quality [9]. In a clinical setting, more than half of patients with chronic pain reported insomnia, and sleep disturbance was significantly associated with pain intensity, sensory pain ratings, affective pain ratings, general anxiety, general depression, and health anxiety [10].

The association between chronic pain and sleep disorders is bidirectional [11]; on the one hand pain leads to sleep disturbance and on the other hand, patients with persistent insomnia often develop chronic pain [4]. Psychological factors, such as mood and pain-related attention [12], and pre-sleep arousal [13] have been shown to play an important role in the interaction between sleep quality and chronic pain. Common mental disorders, such as depression and anxiety often emerge together with chronic pain [2, 3, 14, 15] and with insomnia [16, 17]. When depression and insomnia co-occur in chronic pain patients, they have adverse effects on many pain outcomes [18].

Studies on sleep quality in chronic pain patients are scarce in the Austrian population. The aim of the present analysis was to investigate the association between different dimensions of chronic pain, such as pain intensity, sensory dimension, and affective dimension and different dimensions of sleep quality, such as time before falling asleep, sleep fragmentation, sleep duration, and recovering effects of sleep in subjects suffering from chronic pain. Additionally, it was the aim to evaluate how this association changed by controlling for possible confounders, such as sociodemographic factors, pain characteristics, and physical and psychological quality of life.

## Methods

### Subjects

This analysis is part of a larger study with the primary aim to assess the cost of illness in patients which chronic pain in Austria. Patients from three different hospital-based outpatient clinics were included in the study: the outpatient clinic of the Department of Physical Medicine and Rehabilitation of the Medical University of Vienna, the headache clinic of the Department of Neurology of the Medical University of Vienna and the pain clinic of the Orthopedic Hospital Speising in Vienna. Inclusion criteria were: chronic pain (pain lasting for at least 3 months) at any body site, age between 18 and 65 years, sufficient knowledge of the German language, and currently no cancer and no psychiatric diseases. Exclusion criteria were current cancer treatment or current psychiatric inpatient treatment according to the patients’ report.

### Procedures

Patients were fully informed about the study and the possibility to participate by the treating doctor during a visit to the clinics. In the headache clinic, patients were additionally recruited via mail and asked to participate in the study. Recruitment of study participants lasted from December 2012 to February 2014. All subjects signed an informed consent form to participate in the study. The local ethics committee of the Medical University of Vienna approved the study (EK-number 1624/2012).

### Outcome measures

Location of chronic pain was assessed with a two-sided homunculus diagram, and subjects were asked to show on this drawing the body site or body sites in which they experienced chronic pain. In total, 14 different body locations could be identified. This approach was also used in publications based on the Austrian Health Interview Survey [2]. Pain characteristics were assessed with the short-form McGill pain questionnaire (SF-MPQ) [19]. This questionnaire consists of 15 pain descriptors which are assessed on a Likert scale from 0 (none) to 3 (severe), a visual analogue scale (VAS) for the current overall pain intensity ranging from 0 (no pain) to 10 (most severe pain imaginable) and a present pain intensity (PPI) scale to indicate overall pain intensity by labeling current pain from 0 (no pain) to 5 (excruciating pain). The first 11 items were summed to indicate the sensory dimension of pain (e. g. throbbing and aching), with a possible score from 0 to 33. The next four items were summed to indicate the affective dimension of pain (e. g. fearful and sickening), with a possible score from 0 to 12. The final score for the SF-MPQ was calculated by summing the scores for the sensory dimension, the affective dimension, PPI, and VAS. The total score has a possible range from 0 to 60. For the study, the German version of the SF-MPQ was used [20].

Sleep was assessed with a questionnaire used in a previous study [21]. From this questionnaire, four questions regarding sleep were applied: [1] “how long do you need to fall asleep?”, which could be answered by “up to 30 min”, “up to 1 h” and “longer than 1 h” [2]. “How often is your sleep interrupted due to pain?”, which could be answered by “not at all”, “1–3 times per night” and “3 times per night and more” [3]. “How long do you sleep in total?”, which could be answered by “less than 5 h”, “5–7 h” and “7 h and more” [4]. “Do you feel fresh and recovered after waking?”, which could be answered by “yes” or “no”. Additionally, the use of sedative medication was assessed with the question “do you use prescribed medication for sleep, such as sedatives, antidepressants, anxiolytic drugs or over the counter medication against sleeping problems?”, which could be answered by “yes” or “no”.

As the covariate, health-related quality of life (HRQOL) was assessed by the use of the short-form 12 health survey (SF-12 questionnaire), and summed scores for the dimensions of physical and psychological quality of life were calculated [22]. Further covariates were sex, age, and level of education in three levels: primary education (compulsory school up to the age of 15 years), secondary education (apprenticeship, professional/commercial school, and high school up to the age of 19 years), and tertiary education (university).

Statistical analyses were calculated with IBM SPSS Statistics V21.0. Descriptive statistics were applied to describe the sample characteristics. For this, either mean and standard deviation (SD), where appropriate or numbers (*N*) and percentages (%) are indicated. To describe the association between pain characteristics and sleep parameters, analyses of variance (ANOVA) was applied. Finally, stepwise logistic regression models were calculated with the four dichotomized sleep parameters as the dependent variables and the total score of SF-MPQ as the independent variable. After an initial crude model, in model I adjustment was made for age, sex, and level of education. In model II additional adjustment for duration of chronic pain and the number of painful body sites was made. Finally, in model III, additional adjustments were made for the summed scores for physical and psychological quality of life according to the SF-12. Results are presented as odds ratios (ORs) and 95% confidence intervals (95% CI).

## Results

In total 121 patients with chronic pain were included in the study. The age range was from 23 to 65 years, and almost three quarters were women. Most of the subjects had secondary education, but the proportion with university as the highest education (one fifth) was remarkably high (Table 1). The majority of subjects indicated that the pain had already lasted for longer than 10 years. Most subjects indicated more than one body site with chronic pain. The most common body sites were lower back (57.9% of the patients), neck (51.2%), legs (50.4%), shoulders (44.6%), head (35.5%), hips (32.2%), feet and toes (28.9%), arms (25.6%), upper back (24.8%), hands and fingers (23.1%), and face (20.7%). Mean values for the dimensions of SF-MPQ are listed in Table 1. Approximately one fifth of the subjects indicated that they needed more than I h to fall asleep. The majority reported that they woke up regularly due to pain and one third indicated a sleep duration of less than 5 h. Almost two thirds answered that they perceived their sleep as non-recovering (Table 1). The question regarding the use of sleep medication was answered by 25.6% with “yes” and 72.7% with “no” (1.7% missing).

**Table 1:** Characteristics of the 121 participants (male 32, female 89, mean age 49 ± 9 years) with chronic pain

	Mean or *N*	SD or %
*Level of education*		
Primary	21	17.4
Secondary	74	61.2
Tertiary	26	21.5
SF-MPQ (sensory dimension)	11.2	6.0
SF-MPQ (affective dimension)	4.5	2.9
SF-MPQ (PPI score)	3.3	0.8
SF-MPQ (VAS)	4.0	2.6
SF-MPQ (total score)	22.9	9.4
Duration of chronic pain (years)	15.5	14.5
Number of painful body sites	4.1	2.8
SF-12 (physical sum score)	35.4	10.6
SF-12 (psychological sum score)	45.1	12.2
*Falling asleep*		
Up to 30 min	74	61.2
Up to 60 min	24	19.8
Longer than 60 min	23	19.0
*Sleep fragmentation*		
None	44	36.4
1–3 times per night	57	47.1
More than 3 times	20	16.5
*Sleep duration*		
More than 7 h	18	14.9
5–7 h	66	54.5
Less than 5 h	37	30.6
*Recovering effect of sleep*		
Yes	48	39.7
No	73	60.3

*SF-MPQ* short-form McGill pain questionnaire, *PPI* present pain intensity, *VAS* visual analogue scale, *SF-12* short-form-12 health survey

According to the results of ANOVA, there was a clear association between the affective dimension of pain, the PPI score, the VAS score, the total SF-MPQ score and the duration until falling asleep. There was also a clear association between the affective dimension, the PPI score, and the total score of pain experience with the probability of waking up due to pain. Furthermore, the sensory dimension, the affective dimension, the PPI score, and the total score were significantly associated with the probability of perceiving sleep as recovering. There was no significant association of any pain measure with sleep duration (Table 2).

**Table 2:** Mean values of the different dimensions of the SF-MPQ by different sleep parameters (ANOVA)

	Sensory dimension	*P*-value	Affective dimension	*P*-value	PPI score	*P*-value	VAS score	*P*-value	Total score*	*P*-value
*Falling asleep*		*0.087*		*0.005*		*0.041*		*0.046*		*0.003*
Up to 30 min	10.4		4.0		2.2		3.6		21.1	
Up to 60 min	11.1		4.5		3.3		3.9		22.9	
Longer than 60 min	13.2		6.2		3.7		5.1		28.6	
*Sleep fragmentation*		*0.055*		*0.012*		*0.044*		*0.581*		*0.021*
None	9.5		3.5		3.1		3.8		19.8	
1–3 times per night	12.4		5.3		3.5		4.0		25.1	
More than 3 times	11.5		4.3		3.4		4.5		23.6	
*Sleep duration*		*0.196*		*0.164*		*0.151*		*0.476*		*0.095*
More than 7 h	11.0		4.4		3.2		3.6		22.1	
5–7 h	10.6		4.2		3.4		4.0		22.1	
Less than 5 h	13.5		5.7		3.7		4.5		27.4	
Recovering sleep		0.026		0.007		0.015		0.602		0.009
Yes	9.7		3.6		3.1		3.8		20.1	
No	12.2		5.1		3.5		4.1		24.7	

* differences between the sum of the lines and the presented numbers are due to missing numbers in subdomains of the McGill questionnaire

*SF-MPQ* short-form McGill pain questionnaire, *PPI* present pain intensity, *VAS* visual analogue scale

The binary logistic regression analyses revealed that one point more in the total score of SF-MPQ increased the odds of needing more than 30 min for falling asleep, waking up more than three times due to pain, sleeping less than 5 h, and perceiving the pain as non-recovering, by 6% (crude model in Table 3). Adjusting for socio-demographic factors (model I, Table 3), duration of pain, and number of painful body sites (model II, Table 3) did not clearly alter the association between pain and sleep parameters; however, adjusting additionally for physical and psychological quality of life lowered the odds ratio (OR) and the association was no longer significant; therefore, a modifying effect of the quality of life on the association between pain perception and sleep quality can be assumed (model III, Table 3).

**Table 3:** Association between total SF-MPQ-score (independent variable) and the various sleep parameters (dependent variables). Results of stepwise bivariate logistic regression models

	Crude OR (95% CI)	Model I OR (95% CI)	Model II OR (95% CI)	Model III OR (95% CI)
Falling asleep after more than 30 min	1.06 (1.01–1.10)	1.06 (1.02–1.11)	1.06 (1.02–1.12)	1.05 (0.99–1.11)
Waking up more than three times due to pain	1.06 (1.02–1.11)	1.07 (1.02–1.12)	1.08 (1.02–1.13)	1.06 (1.00–1.13)
Sleep duration < 5 h	1.06 (1.00–1.12)	1.07 (1.01–1.13)	1.07 (1.01–1.14)	1.03 (0.96–1.11)
No perceived recovering sleep	1.06 (1.01–1.11)	1.05 (1.00–1.10)	1.05 (1.00–1.10)	1.01 (0.94–1.06)

Model I: adjusted for age, sex, and level of education

Model II: as model I and additionally adjusted for duration of chronic pain and number of painful body sites

Model III: as model II and additionally adjusted for the sum scores of physical and psychological quality of life (SF-12)

*SF-MPQ* short-form McGill pain questionnaire, *SF-12* short form-12 health survey, *OR* odds ratio, *CI* confidence interval

## Discussion

The results of the present study showed notable sleep disturbances in the sample of patients suffering from chronic pain. The affective dimension of pain and pain intensity were particularly associated with sleep problems. The time needed for falling asleep, frequency of waking up in the night, and experiencing sleep as recovering were sleep dimensions most often influenced by pain, whereas sleep duration was only marginally affected by the perception of pain. Interestingly, the association between sleep quality and pain was not influenced by sociodemographic variables, duration of pain or the number of painful body sites; however, physical and psychological quality of life modified this association, since adjustment for those factors lowered the OR and led to a loss of significance of this association.

In the present study, the majority of the patients with chronic pain were females. This relates to the fact that chronic pain disorders have considerable higher prevalence in females than males [7]; however, adjusting for sex did not affect the association between pain and sleep quality (Table 3), hence the association between pain and sleep quality did not show gender differences. In another study, however, female patients with chronic pain reported poorer sleep quality than males [9]. The majority of the participants in our study reported falling asleep in up to 30 min (Table 1). This result might indicate that falling asleep is not the major sleep difficulty for the majority of chronic pain patients. Nevertheless, the proportion of patients with chronic pain who relatively quickly fall asleep was lower than the proportion (76%) in the general Austrian population [23]. Furthermore, three out of four pain assessment dimensions (i.e. affective dimension, PPI and global pain intensity), as well as the total score of the SF-MPQ, showed significant associations with sleep latency (Table 2). These results emphasize a link between sleep onset latency and intensity of chronic pain and pain-related cognition. In comparison, Tang et al. reported in their study on patients with chronic back pain that affective pain ratings and health anxiety were significant predictors of impaired sleep latency [10]. Sleep fragmentation (1–3 times per night) was reported in almost half of our patients (Table 1). In contrast, in the observation of the general population only 15% described difficulties in sleep maintenance [23]. In the present study, more than half the subjects reported sleeping 5–7 h per day (Table 1). Sleep duration showed no significant associations with the mean values of the different dimensions of the SF-MPQ by the form of the different sleep parameters (Table 2). These results are in accordance with the results of the study conducted by Smith et al. [13], who reported an average sleep duration of 6 h per day in a sample of patients suffering from chronic pain. Nevertheless, while sleep duration did not seem to be impaired in our study, recovering effects of sleep were reported in only a minority of participants (Table 1), and significant associations between non-recovering sleep with the mean values of the different dimensions of the SF-MPQ by the form of the different sleep parameters were found (e.g. sensory dimension, affective dimension, PPI and total score, Table 2).

In our study, the affective dimension of chronic pain (e.g. experiencing pain as tiring exhausting, sickening, fearful, and cruel punishing) showed the highest impact on sleep quality next to pain intensity. This finding underlines the strong effect of psychological factors on the association between pain and sleep. This fact is in line with previous literature, where common mental disorders, such as depression [13, 24–27] and anxiety [24, 26, 27] were highly associated with sleep disturbances in patients with chronic pain.

Sleep disturbance experienced by patients with chronic pain is receiving growing attention as an important factor in the quality of life. In our study, when controlling for the physical and psychological dimensions of the quality of life, the association between pain parameters and sleep quality was lowered and not significant any more. This means that at a given level of quality of life we found no significant association between pain dimensions and sleep dimensions. This can also be interpreted that physical and psychological quality of life mediates the association between pain and sleep quality. Quality of life seems to have a central role for patients with chronic pain, which is in line with a previous Austrian study [28].

Despite the observational nature of our study, some possible clinical implementations should be discussed. Our results might support the assumption that sleep and pain have a bidirectional and reciprocal relationship; therefore, clinicians who manage patients with chronic pain should focus on interventions that relieve pain, as well as on assessing and treating sleep disturbance. This is, however often only addressed as a secondary concern [29]. In addressing sleep quality in patients with chronic pain, it has to be considered that the effect of various medication groups on sleep in chronic pain patients are inconclusive. While non-steroidal anti-inflammatory drugs are sleep neutral, antidepressants and opioids can have positive as well as negative effects on sleep [30]. Finally, our results underline the importance of psychological factors, such as mood, anxiety, depression, and quality of life in the association between pain and sleep quality. These factors should be routinely addressed in the management of patients with chronic pain in terms of assessment, monitoring and defining treatment goals; therefore, a multidisciplinary approach will often be required in order to obtain more comprehensive improvements for patients in medical, functional, and social contexts.

The present study has some limitations. Firstly, it relies solely on self-reported measures of sleep disturbance; therefore, subjective sleep problems might be overestimated by the patients. Nevertheless, a study by O’Donoghue et al. showed that participants with chronic low back pain demonstrated significantly poorer sleep, irrespective of the kind of sleep evaluation (objective or subjective) [31]. Furthermore, the cross-sectional design did not permit any conclusions regarding the direction of the relationship between pain and sleep. It cannot be determined from the results whether sleep disturbance is only a marker for nociceptive processes or whether insomnia might also contribute to hyperalgesia. Additionally, it has to be mentioned that it was only the secondary aim of the study to evaluate the association of pain and sleep quality. The sample was set for the primary aim, to assess the cost of illness in patients with chronic pain. The size of the sample is also a limitation, yielding a low power for some statistical calculations. Finally, we did not assess the clinical diagnoses of common mental disorders, such as depression, anxiety, or stress-related disorders, only the mental dimension of quality of life, which limits conclusions about the mediation of psychological factors.

## Conclusion

Various sleep problems are significantly associated with pain in patients suffering from chronic pain. Physical and psychological dimensions of quality of life notably influence both pain perception and sleep quality and therefore modify the association between pain perception and sleep quality. Because comorbid sleep problems and pain have been related to higher disability, the need to improve sleep quality among patients with chronic pain, and to reduce pain among patients with insomnia, should be an important part of future research.
